# Total Nitrogen Concentrations in Surface Water of Typical Agro- and Forest Ecosystems in China, 2004-2009

**DOI:** 10.1371/journal.pone.0092850

**Published:** 2014-03-25

**Authors:** Zhiwei Xu, Xinyu Zhang, Juan Xie, Guofu Yuan, Xinzhai Tang, Xiaomin Sun, Guirui Yu

**Affiliations:** Key Laboratory of Ecosystem Network Observation and Modeling, Institute of Geographic Sciences and Natural Resources Research, Chinese Academy of Sciences, Beijing, China; University of Vigo, Spain

## Abstract

We assessed the total nitrogen (N) concentrations of 28 still surface water (lake and pond), and 42 flowing surface water (river), monitoring sites under 29 typical terrestrial ecosystems of the Chinese Ecosystem Research Network (CERN) using monitoring data collected between 2004 and 2009. The results showed that the median total N concentrations of still surface water were significantly higher in the agro- (1.5 mg·L^−1^) and oasis agro- ecosystems (1.8 mg·L^−1^) than in the forest ecosystems (1.0 mg·L^−1^). This was also the case for flowing surface water, with total N concentrations of 2.4 mg·L^−1^, 1.8 mg·L^−1^ and 0.5 mg·L^−1^ for the agro-, oasis agro- and forest ecosystems, respectively. In addition, more than 50% of the samples in agro- and oasis agro- ecosystems were seriously polluted (>1.0 mg·L^−1^) by N. Spatial analysis showed that the total N concentrations in northern and northwestern regions were higher than those in the southern region for both still and flowing surface waters under agro- and oasis agro- ecosystems, with more than 50% of samples exceeding 1.0 mg·L^−1^ (the Class III limit of the Chinese National Quality Standards for Surface Waters) in surface water in the northern region. Nitrogen pollution in agro- ecosystems is mainly due to fertilizer applications, while the combination of fertilizer and irrigation exacerbates nitrogen pollution in oasis agro- ecosystems.

## Introduction

Nitrogen (N) pollution, leading to eutrophication of inland waters, has resulted in an increase in global algal biomass and photosynthesis, such that primary production is approximately 60% higher than expected background levels in lakes [1], streams and rivers [2]. As a major contributor to eutrophication of water bodies, non-point losses of N (e.g. in runoff) have received particular attention. N pollution causes water eutrophication, which disrupts ecology and causes, among other problems, toxic algal blooms, loss of oxygen, loss of biodiversity (including species that are important for commerce and recreation) [3]–[5]. Eutrophication can also seriously affect our ability to use water for drinking, industry, agriculture, recreation, and other purposes.

Total N concentrations in surface water have been classified in China and other countries to control water eutrophication and improve water quality. To identify at-risk surface water bodies and protect them from eutrophication, the US EPA developed guidelines, which state that N concentrations should not exceed 0.3 mg·L^−1^ in streams and rivers or 0.1 mg·L^−1^ in lakes and reservoirs [6]. Water has been divided into 14 distinct aggregate nutrient ecoregions in the U.S. according to total P and total N concentrations, chlorophyll *a* and turbidity. In China surface water has been divided into five categories according to the Chinese National Quality Standards for Surface Water. Water categorized as class I to III can be used as drinking water, while class IV and V water is only suitable for industrial and agricultural uses. The total N values for categories I – V are < 0.2 mg·L^−1^, 0.2 – 0.5 mg·L^−1^, 0.5 – 1.0 mg·L^−1^, 1.0 – 1.5 mg·L^−1^ and 1.5 – 2.0 mg·L^−1^, respectively [7].

In China, stream total N concentrations have tended to increase since the 1980s as a consequence of demographic, industrial and agricultural development [8], [9]. Rivers and lakes in the Taihu Lake region are polluted to varying degrees by N [10], with 80% of samples having concentrations exceeding 1.0 mg·L^−1^, meaning that the water is only suitable for industrial, agricultural and landscape uses according to the Chinese National Quality Standards for Surface Water [11]. Cai et al. [12] reported that eutrophication of lakes is serious in southern China, but that it is worse across a large part of northern China. Rural rivers in eastern China are severely polluted [13]. As a general rule, water pollution in China tends to intensify from tributaries to the main stems of river systems, from urban to rural areas, from surface water to groundwater and from the regional to the basin scale [12].

Natural and anthropogenic sources both contribute to surface water N inputs [14], [15]. Now that point-source pollution has been controlled effectively, non-point source pollution, especially that which results from agricultural land management, has become the main influence on surface water and is an important environmental issue worldwide [16], [17]. Crop production is by far the largest cause of human alteration of the global N cycle. Global industrial N fixation for fertilizers has increased rapidly to 80×10^6^ Mg·year^−1^ [18]. In the United States (US) and Europe, only 18% of the N input in fertilizer is removed from farms in produce, leaving behind, on average, 174 kg·ha^−1^·year^−1^ of surplus N [19], [20]. This surplus may accumulate in soils, from where it may be either eroded and transported to surface water, leached to surface and ground water, or lost to the atmosphere [18]. Inorganic fertilizers now contribute 80 Tg N year^−1^ to the environment, while 32 – 45 Tg year^−1^ of inorganic fertilizers find their way into freshwater (from leaching and erosion) [21].

Fertilizer consumption has grown rapidly in China since 1978, and China recently became the biggest producer and consumer of N fertilizer in the world. The average annual application rate of N in China gradually increased to 130 kg·ha^−1^ in 1985, then rapidly increased to 236 kg·ha^−1^ in 1995 and then to 262 kg·ha^−1^ in 2001 [22]. However, less than half of the fertilizer N applied in China is taken up by crops [23]. The losses of total N through runoff and leaching were 5.6 – 9.0 kg·ha^−1^ from paddy fields and 12.5 – 19.5 kg·ha^−1^ from upland fields, accounting for 2.6 – 4.2% and 7.6 – 12.3% of the total applied N [24]. It is estimated that as much as 0.644 Tg of total N is leached, accounting for 2.8% of China's total N application [24].

Because of the increasing incidence of surface water N pollution, many countries are beginning to be concerned about sustainable water management. The European Union has implemented the Water Framework Directive, which aims to recover good status in water resources by 2015 [25]. Canada (both at the national and provincial levels) and the US have begun to take an effective and integrated approach to land-use management with respect to protection of drinking water sources. On a national level, Canada has established aboriginal water systems, while at the provincial level; British Columbia has established a water policy, similar to that developed by the US Environmental Protection Agency [26]. The Chinese government has published many water environmental protection measures such as the Water Pollution Prevention Program of Yangtze River basin (2011–2015) and other measures for important river basins (Yellow River, Huaihe River, Weihe River, Liaohe River, Haihe River, Songhuajiang River, Chaohu and Dianchi) [27]. Until now however, there have been few national scale assessments of N concentrations in surface water in China.

Using the Chinese Ecosystem Research Network (CERN) as the monitoring framework, total N concentrations at 28 still, and 45 flowing, surface water monitoring sites of 28 CERN monitoring stations were assessed from 2004 to 2009. The aims of this study were: (1) to assess surface water total N pollution under agro-, oasis agro- and forest ecosystems and (2) to identify sites vulnerable to N pollution in agro- and oasis agro- ecosystems. Findings from this study will provide key information that will be useful in controlling N pollution in surface water in China.

## Materials and Methods

### 2.1 Ethics statement

The authors declare that no specific permits were required for the described field studies. The authors also declare that no specific permissions were required for these locations/activities. Locations are not privately-owned or protected in any way. We confirm that this study did not involve endangered or protected species and no protected species were sampled during the monitoring campaign.

### 2.2 Monitoring sites

**Figure 1:**
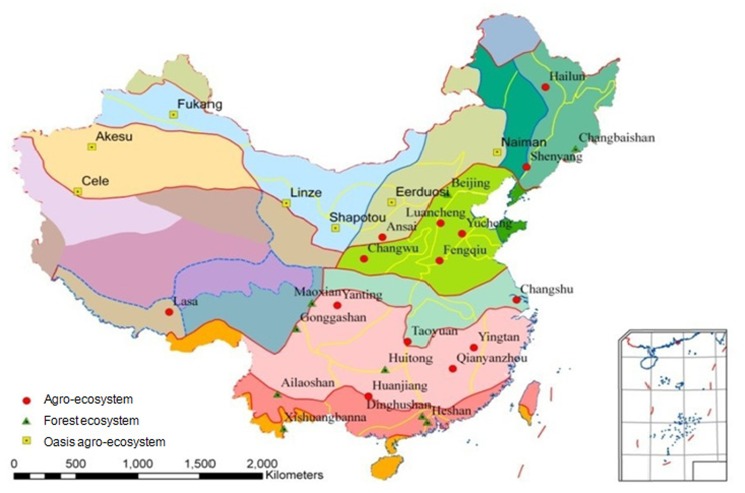
Distribution map of total nitrogen monitoring sites in agro- , oasis agro- and forest ecosystems of the Chinese Ecosystem Research Network (CERN).

Surface water monitoring of the CERN focuses on assessing the effects of typical terrestrial ecosystems, i.e. agro-, oasis agro- and forest ecosystems, on water quality. Twenty-eight monitoring stations of the CERN were chosen to represent typical agricultural, oasis agricultural and forest ecosystems, geology, soils, and land use types found across a wide range of climatic zones in China (Figure 1). The annual average rainfall of the monitoring ecosystems (80°43′39″-133°18′03″E, 18°13′01″-47°27′15″N) varied from approximately 35 mm (Cele) to 1956 mm (Dinghushan), while the annual average temperature ranged from 1.5°C (Hailun) to 21.8°C (Xishuangbanna).

The 12 agro- ecosystems were located in (1) humid and sub-humid regions in the temperate zone of northeastern China (Hailun, Shenyang) and the warm temperate zone of northern China (Luancheng, Yucheng, Fengqiu), and (2) the Loess Plateau (Ansai and Changwu), (3) humid areas in the sub-tropical zone in the Yangtze River Delta (Changshu) and southern China (Huanjiang, Qianyanzhou, Yanting, Taoyuan,Yingtan), respectively (Figure 1). Based on geographical and climatic zones, we designated Hailun, Shenyang, Luancheng, Yucheng, Fengqiu, Ansai and Changwu agro- ecosystems as the northern group, while the Changshu, Huanjiang, Qianyanzhou, Yanting, Taoyuan and Yingtan agro- ecosystems were designated as the southern group. Land use in these agro- ecosystems was mainly crop growing, including wheat, maize, soybean, rice and cotton (Table 1).

**Table 1:** TN concentrations in still and flowing surface water under agro- and oasis agro- ecosystems.

Spatial Regions	Station	Mean Precipitation (mm)	Soil type	Land use	N application rate (kg·ha^−1^year^−1^)	Still surface water				Flowing surface water			
						n	Median	Max	>1.0 mg·L^−1^ frequency(%)	n	Median	Max	>1.0 mg·L^−1^ frequency(%)
North	Ansai	500	Loessial Soil	Soybean-Millet	120	—	—	—	—	56(1)	2.8	13.8	96
	Changwu	584	Loessial Soil	Maize-Wheat	345	24(1)	3.0	9.7	100	17(2)	1.6	3.9	65
	Fengqiu	597	Fluvo-aquic Soil	Maize- Wheat	345	—	—	—	—	57(1)	3.3	39.1	96
	Hailun	500-600	Black Soil	Wheat-Maizerotation	120	—	—	—	—	19(1)	57.0	84.8	100
	Shenyang	650-700	Aquic brown Soil	Maize	75	13(1)	0.8	9.4	23	11(1)	0.3	0.4	0
	Yucheng	582	Fluvo-aquic Soil	Maize-Wheat	510	—	—	—	—	75(2)	5.4	27.8	91
South	Changshu	1038	Red Soil	Paddy-Wheat	466	46(2)	1.9	7.9	80	96(1)	1.8	12.3	75
	Huanjing	1389	Calcareous soil	Maize-Soybean	—	5(1)	0.6	9.0	20	10(2)	0.3	10.3	20
	Qianyanhzou	1542	Red Soil	Paddy-Paddy	320	19(2)	1.0	7.3	53	9(1)	0.9	2.0	44
	Yanting	826	Purple Soil	Maize -Wheat	300	32(1)	1.7	4.5	84	—	—	—	—
	Taoyuan	1450	Red Soil	Paddy-Paddy	270	9(2)	1.0	4.4	44	20(2)	0.9	8.0	40
	Yingtan	1785	Red Soil	Peanut	150	44(2)	0.6	3.0	9	43(6)	0.8	34.8	35
Northwest	Akesu	45.7	Aeolian sandy soil	Cotton	160	15(2)	0.9	1.6	40	7(1)	0.8	1.8	43
	Cele	35	Aeolian sandy soil	Cotton-Maize rotation	468	18(1)	1.0	3.2	39	19(1)	2.8	13.4	84
	Eerduosi	348.3	Aeolian sandy soil	Cotton-Maize rotation	—	4(1)	1.0	1.2	50	21(1)	2.2	4.3	95
	Fukang	164	Aeolian sandy soil	Cotton-Maize rotation	275	9(1)	1.5	17.4	89	8(1)	1.6	91.0	63
	Linze	117	Aeolian sandy soil	Wheat–Maize rotation	122	8(2)	23.3	69.5	100	10(1)	12.2	72.0	100
	Naiman	340-450	Aeolian sandy soil	Wheat-Maize rotation	207	8(1)	1.4	8.3	75	14(2)	0.9	1.3	36
	Shapotou	180-220	Aeolian sandy soil	Wheat-Maize rotation	256	10(1)	11.5	43.0	100	—	—	—	—

Note: The “n” values represent the number of sampling sites and the monitoring sites (within brackets). “-”s illustrate that no detection data were available.

Seven oasis agro- ecosystems (Akesu, Cele, Eerduosi, Fukang, Linze, Naiman, Shapotou) were located in the warm temperate zone of northwest and northern China in arid and semi-arid areas. The oasis agro- ecosystem is unique and found only in arid agricultural areas. Unlike agro- ecosystems in other areas represented in this study, crop cultivation depends heavily on irrigation. Land use in the oasis agro- areas was mainly cotton, maize and wheat growing (Table 1). Therefore, we classified these ecosystems as an independent group and called it oasis agro- ecosystem. We designated these seven oasis agro- ecosystems as the northwestern group.

Total N concentrations in surface water in forest ecosystems were assessed. The forest ecosystems were located along the north-south transect of eastern China (Figure 1), and were representative of native and secondary forests and were free from human fertilization and irrigation activities.

### 2.3 Monitoring and analysis methods

CERN surface water quality samples were collected according to the Water Monitoring Protocol of the Chinese Ecosystem Research Network [28]. 530 samples were collected from the still surface water sites, and 703 samples were collected from the 45 flowing surface water monitoring sites. The monitoring frequency ranged from 2 to 12 times per year, with sampling distributed evenly through the wet and dry seasons. The maximum sampling frequency was monthly (Ansai, Fengqiu, and Changshu), and the minimum sampling frequency was twice a year (for most of the other monitoring ecosystems), in both the dry and wet seasons. Water samples were analyzed at the Chinese Science Academy’s laboratory following standard protocols and methods [28]. Total N was determined by spectrophotometry after potassium persulfate digestion. Information about crop rotation, soil type and fertiliser application rate was recorded for each sampling event at each monitoring station.

### 2.4 Statistical analysis

Data were analyzed with Matlab 7.11.0 (Massachusetts, USA). A Lilliefors test was conducted to test the normality of the data. Data were not normally distributed, so the nonparametric Kruskal-Wallis test was used to test for differences between the median total N concentrations for data grouped by ecosystem type (for agro-, oasis agro- and forest ecosystems) and geographical region (southern, northern and northwestern) for agro- and oasis agro- ecosystems. Where there were differences between data groups, the Kruskal-Wallis test was combined with a multi-comparison method in Matlab R 2010b to determine which groups were different (*p* < 0.05). We used linear regression to test for relationships between total N concentrations and soil N application rates using SPSS 19.0 for Windows. We used *p* < 0.05 as the significance level. We used Origin 8.0 software for box plots.

1.0 mg·L^−1^ (Class III limit of the Chinese National Quality Standards for Surface Waters) was used as the guideline to assess the exceedance frequency of total N concentrations at the monitoring sites in agro-, oasis agro- and forest ecosystems. Sites with high exceedance frequencies were identified as total N vulnerable zones.

## Results

### 3.1 Total N concentrations under different ecosystems

**Figure 2:**
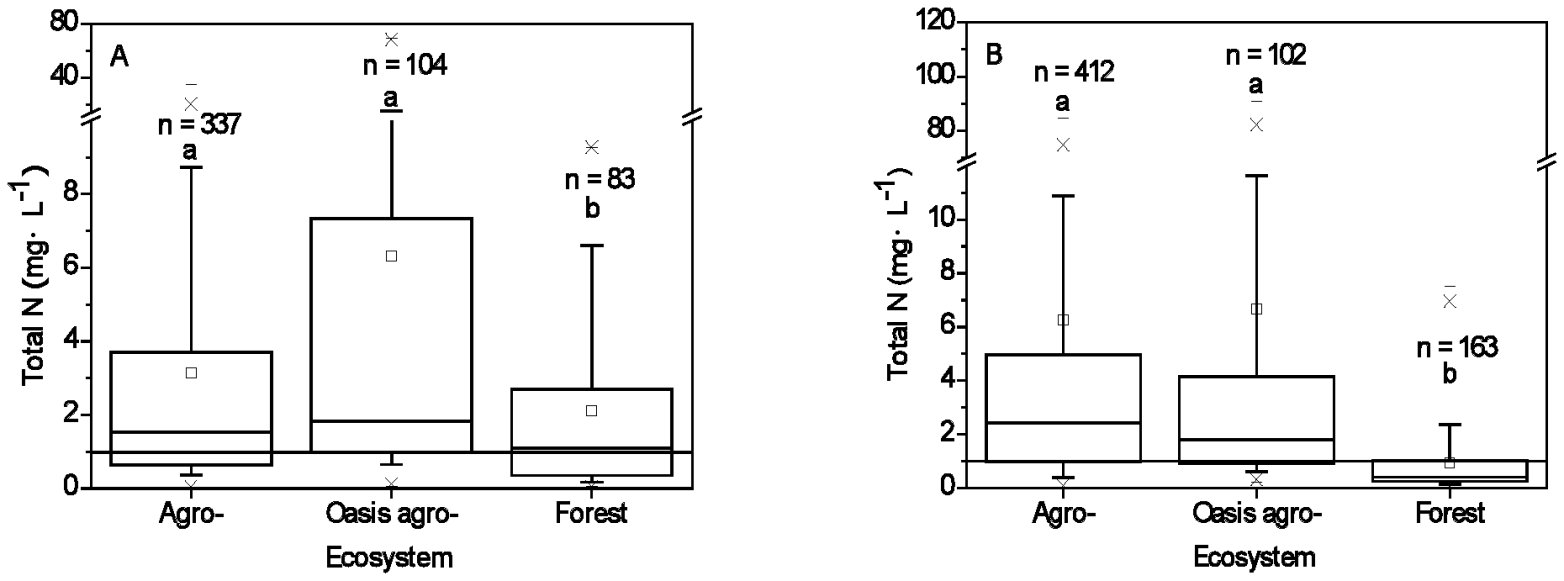
Total nitrogen concentrations in still surface water (A) and flowing surface water (B) of the agro-, oasis agro- and forest ecosystems between 2004 and 2009. Box plots illustrate the 25^th^, 50^th^, and 75^th^ percentiles, the whiskers indicate the 10^th^, 90^th^ percentiles, the “-”s indicate the maximum and minimum percentiles, the “×”s indicate the 1^th^ and 99^th^ percentiles, the “□”s indicate the mean values. Box plots labeled with different letters (a, b) indicate that differences in median values among the three ecosystem types are significant at *p* < 0.05. “—” illustrate the Class III guideline of 1.0 mg·L^−1^ for total nitrogen according to the national quality standards for surface waters of China.

Total N concentrations in the agro- and oasis agro- ecosystems were significantly higher than in the forest ecosystems. The typical total N concentrations (10^th^ and 90^th^ percentiles in box plots) in the agro-, oasis agro- and forest ecosystems ranged between 0.4 – 8.7 mg·L^−1^, 0.7 – 15.2 mg·L^−1^, and 0.2 – 6.6 mg·L^−1^, respectively for still surface water (Figure 2(A)), and 0.4 – 10.9 mg·L^−1^, 0.6 – 11.6 mg·L^−1^, and 0.2 – 3.3 mg·L^−1^, respectively for flowing surface water (Figure 2(B)). The median total N concentrations of still and flowing surface water in forest ecosystems were 1.1 mg·L^−1^ and 0.5 mg·L^−1^, respectively. These concentrations were significantly lower than those of still (1.5 mg·L^−1^ and 1.8 mg·L^−1^, respectively) and flowing surface water (2.4 mg·L^−1^ and 1.8 mg·L^−1^, respectively) in the agro- and oasis agro- ecosystems (*p* < 0.05).

The surface water in agro- and oasis agro- ecosystems was seriously polluted by N. The median concentrations of total N under the agro- and oasis agro- ecosystems exceeded 1.0 mg·L^−1^ [7] (Figure 2), indicating that more than 50% of surface water samples were heavily polluted by N in these ecosystems.

The total N concentrations in surface water were higher in Beijing, Huitong, Heshan and Dinghushan forest ecosystems than the other forest ecosystems. The exceedance frequencies (> 1.0 mg·L^−1^) of flowing surface water total N concentrations in Huitong and Dinghushan were about 100%, which indicates that surface water in some forest ecosystems is eutrophic. The water quality standard was not exceeded for either still or surface water in Gongga, Ailao and Xishuangbanna (Table 2).

**Table 2:** TN concentrations and background information of still and flowing surface water under the 9 forest ecosystems.

Ecotype	Station	Altitude(m)	Mean Precipitation (mm)	Soil type	Vegetation	Still surface water				Flowing surface water			
						n	Median	Max	>1.0 mg·L^−1^ frequency (%)	n	Median	Max	>1.0 mg·L^−1^ frequency(%)
Humid, Sub- humid Areas in Temperate Zone	Changbai	740	695	Dark Brown soil	Broad-leaved korean pine forest	—	—	—	—	9(1)	1.0	1.4	44
Humid, Sub-humid Areas in Warm Temperate Zone	Beijing	1248	612	Mountain brown soils	Man-made Pinus tabulaeformis Forests	—	—	—	—	9(1)	1.6	5.7	56
Humid Areas in North Sub- tropical Zone	Maoxian	1826	825	Cinnamon soil	Warm temperate coniferous forest	—	—	—	—	8(1)	0.7	1.0	13
	Gongga	2950	1974	Podzolie brown taiga soils	Sulbalpine dark coniferous forest	—	—	—	—	54(7)	0.3	0.8	0
	Huitong	541	1079	Yellow soil	Broad-leaved Trees Mixed Forests	11(1)	1.5	3.5	100	13(1)	1.8	3.7	92
Humid Areas in South Sub- tropical Zone	Ailao	2481	1880	Yellow Brown soil	Subtropical mid mountain humid evergreen broadleaved forest	18(1)	0.3	0.9	0	19(1)	0.2	0.5	0
	Heshan	90	1927	Ferrisols	Acacia mangium pure forests	12(1)	0.8	3.3	42	19(1)	1.6	7.5	63
	Dinghu	90	1700	Lateritic Red Soil	Broad-leaved Trees Mixed Forests	30(3)	5.2	9.3	100	10(1)	1.3	7.0	70
Humid Areas in tropical Zone	Banna	560	1539	Red soil	Tropical seasonal rain forest	12(1)	0.3	0.5	0	23(1)	0.2	0.9	0

Note: The “n” values represent the number of sampling sites and the monitoring sites (within brackets). “-”s illustrate that no detection data were available.

### 3.2 Total N concentrations of agro- and oasis agro- ecosystems in different regions

**Figure 3:**
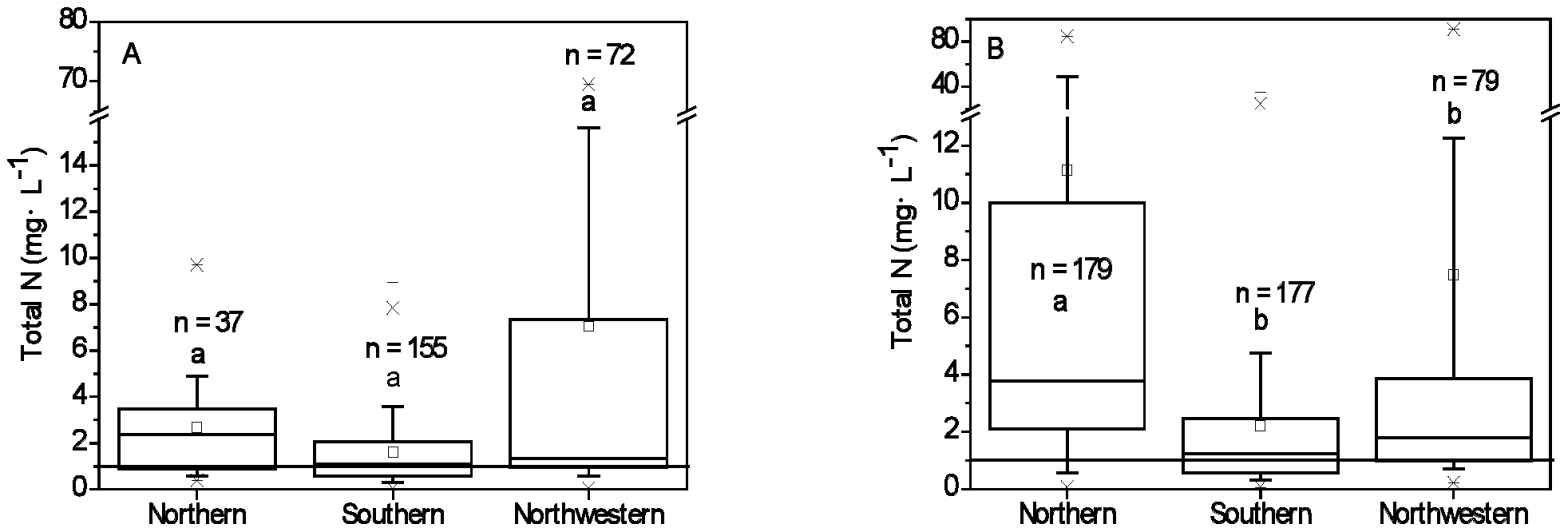
Total nitrogen concentrations of still surface water (A) and flowing surface water (B) under agro- and oasis agro- ecosystems in northern, southern and northwestern areas. The illustrations about Box plots are the same as those in figure 2.

The surface water total N concentrations in northern and northwestern regions in China were higher than those in the southern region. The typical total N concentrations of still surface water in northern, southern and northwestern regions ranged from 0.6 – 4.3 mg·L^−1^, 0.3 – 3.5 mg·L^−1^, 0.6 – 15.6 mg·L^−1^ (Figure 3(A)) and 0.9 – 39.1 mg·L^−1^, 0.3 – 4.4 mg·L^−1^, 0.8 – 12.2 mg·L^−1^ for flowing surface water (Figure 3(B)). There were no significant differences between total N concentrations in still surface water for the different regions. However, the total N concentrations in flowing surface water in the northern region (3.8 mg·L^−1^) were significantly higher than those in the southern (1.2 mg·L^−1^) and northwestern regions (1.8 mg·L^−1^) (*p* < 0.05).

Surface water in northern, southern and northwestern regions showed varying levels of N pollution, with median total N concentrations of still and flowing surface water all exceeding 1.0 mg·L^−1^ [7] (Figure 3). Results indicate that more than 50% of surface water samples were heavily polluted by N in northern agro- ecosystems, especially flowing surface water samples. The median total N concentrations for surface water in the southern and northwestern regions exceeded 1.0 mg·L^−1^ [7]. About 50% of surface water samples were seriously polluted by N in northwestern oasis agro- ecosystems.

The total N concentrations varied by station. The flowing surface water total N concentration was highest in the Hailun agro- ecosystem located in northern China, with a median value of 57.0 mg·L^−1^ (Table 1). The flowing surface water total N concentrations in the northern agro- ecosystems, except Shenyang, all exceeded 1.0 mg·L^−1^. Exceedance frequencies at Ansai, Fengqiu, Hailun and Yucheng were 90 – 100%. In addition, the still surface water at Changwu was heavily polluted by N and had an exceedance frequency of 100%.

In southern agro- ecosystems, the two highest still and flowing surface water total N concentrations occurred in Changshu, the median values of which were 1.9 mg·L^−1^ (still) and 1.8 mg·L^−1^ (flowing). At this station about 80% of total N concentrations in still surface water and 75% of total N concentrations for flowing surface water exceeded 1.0 mg·L^−1^ (Table 1). The still and flowing surface water total N concentrations of other stations (Huanjiang, Qianyanzhou, Taoyuan, Yingtan) were about 1.0 mg·L^−1^, and the exceedance frequencies varied from 20% to 50%.

The total N concentrations in the northwestern oasis agro- ecosystems were highest in Linze and Shapotou. The still and flowing surface water median total N concentrations were 23.3 mg·L^−1^ and 12.2 mg·L^−1^, respectively, in Linze, while for still surface water in Shapotou, the median total N concentration was 11.5 mg·L^−1^. The total N concentrations all exceeded 1.0 mg·L^−1^ in these two stations, indicating that the surface water was heavily polluted by N (Table 1).

### 3.3 Correlations between total N concentrations and N fertilizer application rates in agro- and oasis agro- ecosystems

**Figure 4:**
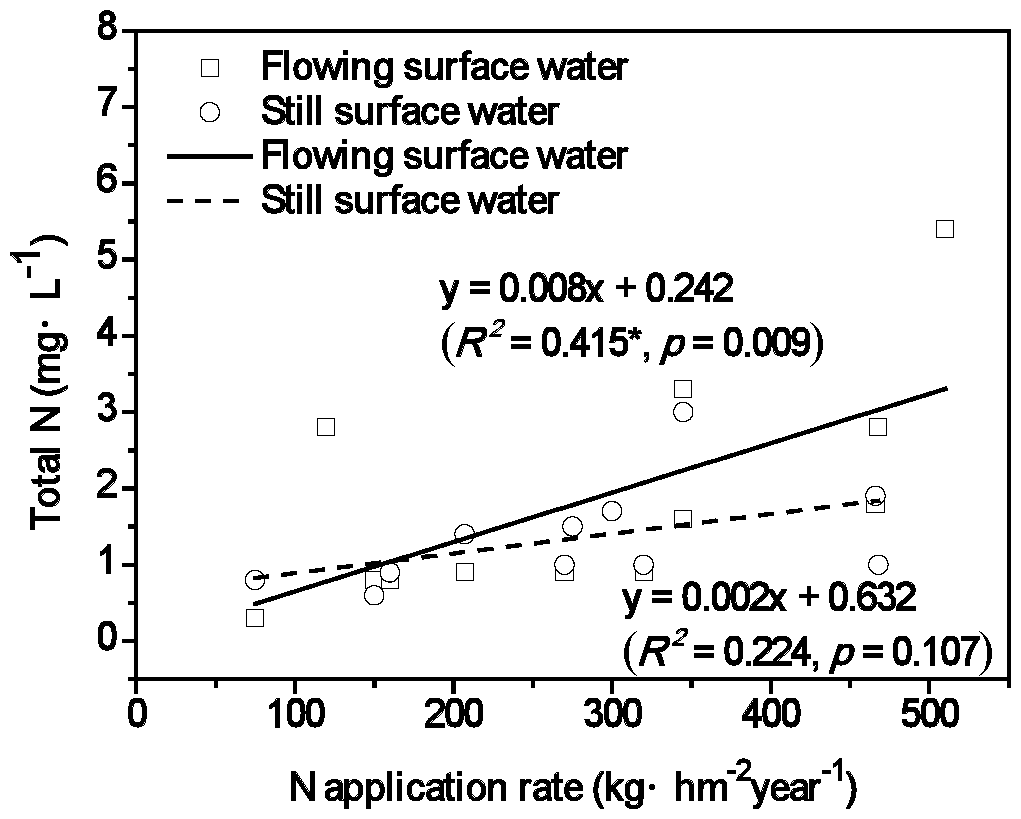
Correlations of total nitrogen concentrations and soil N fertilizer application rates in agro- and oasis agro- ecosystems (*p* < 0.05, n = 15). The total nitrogen concentrations in this figure were the median values of agro- and oasis agro- ecosystems.

Correlation analysis between the surface water total N concentrations and N application rates showed that the flowing surface water total N concentrations and N application rates were significantly correlated (*r* ^2^ = 0.415, *p* = 0.009) (Figure 4). Results indicated that total N concentrations tended to increase as N application rates increased. While there was a similar trend for still surface water and N application rates, the correlation was not significant (*r* ^2^ = 0.225, *p* = 0.107).

## Discussion

### 4.1 Surface water nitrogen pollution in national and international

The median surface water total N concentrations under agro- and oasis agro- ecosystems of the CERN were lower than those in the Tai Lake region (6.4 mg·L^−1^) [29], but similar to those in surface water in northern China [30], [31]. Compared with studies in other countries, the total N concentrations for this study were higher than those of surface water for a Japanese agro- ecosystem [32], but similar to the total N concentrations of the Calapooia River Basin in a Western Oregon agro- ecosystem, where they ranged from 0.5 to 43 mg·L^−1^ [33]. Generally, total N concentrations in northern and northwestern regions were higher than in the southern region of China. A spatial assessment of lakes in China from 1990 to 2010 showed that total N concentrations decreased with rising latitude, but were not related to longitude [12]. Different farming practices, irrigation practices and crop rotations may influence N leaching [34]. For instance, total N losses due to leaching were 9.875 kg·ha^−1^ in the wheat growing season, but were only 1.868 kg N·ha^−1^ in the rice growing season [29]. Li et al [35]. However, reported that nitrate-N concentrations in surface water from rice growing land were significantly higher than those in corn land. In addition, the dilution effect of precipitation could decrease the total N concentrations in the southern region, as precipitation is much higher than in the northern region.

The total N concentrations in surface water under the forest ecosystems were lower than in agro- and oasis agro- ecosystems. This result is similar to what was observed for surface water total phosphorus concentrations in the same study area [36]. Larned et al. [37] also indicated that dissolved N concentrations in pastoral and urban ecosystems were 2 – 7 times higher than in native and plantation forest ecosystems. Total N concentrations were higher in Chinese typical forest ecosystems than in surface water in Japanese forest ecosystems, where they ranged from 0.01 to 1.3, with a median value of 0.14 mg·L^−1^ [38]. In the US, total N concentrations in surface water in northern Californian national forests ranged from 0.03 to 0.1 mg·L^−1^ [39]; these concentrations are also lower than those in China.

Increasing N concentrations have been observed in many watersheds in the US, Europe, New Zealand and Canada because of land use change, atmospheric deposition, fertilizer application and burning fossil fuels [40]–[43]. Total N at the Konza Prairie Biological Station (Konza), located in the Flint Hills region of the Great Plains in Kansas, increased from 0.4 mg·L^−1^ to 1.2 mg·L^−1^ due to land use change [44]. There are concerns about N exports from streams in the US midwest because of excessive nutrient enrichment and eutrophication [45], [46]. In agro- ecosystems in Baltimore, the annual total N exports were about 30 kg N ha^−1^ during 2005 [47]. The annual total N export rates from the world's rivers in different geographical areas between 76 °N and 43 °S ranged from 1 – 20 630 kg N ha^−1^ year^−1^ [48].

### 4.2 Factors influencing TN in surface water

Climate, hydrology, soil properties, geomorphology, topography, soil cover and land use are the main factors that influence nutrients in stream water [49]-[51]. As we were restricted by the sampling frequency, we did not explore relationships between mean annual precipitation and total N in surface waters. However, a significant inverse relationship between total N concentrations and precipitation was reported by a study which examined seasonal variation of total N in the Beijing urban ecosystem [52]. Land use change can influence N concentrations. The N concentrations in rural residential areas and cultivated land were much higher than those in grass and forest ecosystems [53], [54]. Kvítek et al. [55] reported that, as ploughed land in the catchment increased, nitrate contamination of surface water also increased. Studies of N pollution in the US and the Netherlands showed that 60% – 80% of N came from agricultural non-point sources [56]–[58]. Alvarez-Cobelas [48] also reported that exports of total N were four times higher from catchments dominated by crops than from forested catchments.

Nitrogen fertilizer, as a non-point source pollutant, is a critical source of pollution in agro- and oasis agro- ecosystems. Surface water N pollution in agro- ecosystems was closely related to agricultural activity. China surpassed the US and the European Union in its production and use of N fertilizers in approximately 2000. However, less than half of the fertilizer N applied in China was taken up by crops [23]. The rest was largely lost to the environment in gaseous (NH_3_, NO, N_2_O and N_2_) or dissolved (NH_4_^+^ and NO_3_^−^) forms [59], [60]. Leaching is the most important pathway for water eutrophication and poor N fertilizer management could lead to N leaching into the drainage water through run off and drainage [61], [62]. In spite of considerable controls on point source pollution, water quality standards have not reached the criterion of the US and European countries due to the increasing contribution of non-point pollution [63]. In general, the waters under the agro- ecosystems have been widely polluted by total N, while only been polluted by total P in a few agro- ecosystem sites [36]. Therefore, the surface water eutrophication under the agro- ecosystems was P limited, which indicated that N fertilizer should generally be reduced and P fertilizer should only be controlled in the P polluted areas of the agro-ecosystems.

Soil textures differ according to location; therefore, losses of N fertilizer through leaching, drainage and runoff are also variable [64]. The relatively high total N concentrations in surface water in northwestern regions may be attributed to soil types. Studies in France have shown that leached N varied from 31 mg·L^−1^ in deep loamy soils to 92 mg·L^−1^ in shallow sandy soils [34]. Soils in the northwestern region of China are mainly aeolian sandy soils, and have low water-holding capacity, meaning that N fertilizer is likely to be lost from soil to surface water. Combined with frequent irrigation, serious soil losses in this region may contribute to the high total N concentrations [65].

Atmospheric N deposition may be an important source of surface water N [66]. While its effects may be weaker than other factors [69], it may still aggravate water eutrophication and affect ecosystem stability [67], [68]. The 9 forest ecosystems in our study showed eutrophication tendencies, especially the Beijing, Huitong, Heshan and Dinghushan forest ecosystems. The reactive nitrogen (N_r_) species emitted during fossil fuel combustion, have resulted in some of the most pronounced air pollution ever recorded in China, and the increased N_r_ emissions may have influenced atmospheric N deposition near the study areas. Research has shown that N deposition in Dinghushan and Beijing exceeds 30 kg·ha^−1^ year^−1^. These rates are higher than those recorded for the other forest ecosystems in China [70]–[72]. Therefore, the higher atmospheric N deposition may contribute to the higher total N concentrations in the Beijing and Dinghu forest ecosystems.

## Conclusions

The total N concentrations of surface water under agro- and oasis agro- ecosystems were much higher than those under forest ecosystems. About 50% of median total N concentrations exceeded 1.0 mg·L^−1^. There was an obvious spatial pattern in surface water total N concentrations, with much higher total N concentrations in the northern region of China, especially in flowing surface water. The surface water of some agro- ecosystems (Ansai, Changwu, Fengqiu, Hailun) and oasis agro- ecosystems (Cele, Linze, Eerduosi) were severely polluted by N with exceedance ( > 1.0 mg·L^−1^) frequencies greater than 50%. Fertilizer applications were the main source of N pollution in the flowing surface water in agro- and oasis agro- ecosystems.
